# No difference in hypertension prevalence in smokers, former smokers and non-smokers after adjusting for body mass index and age: a cross-sectional study from the Czech Republic, 2010

**DOI:** 10.1186/s12971-015-0049-4

**Published:** 2015-08-11

**Authors:** Alexandra Pankova, Eva Kralikova, Keely Fraser, Jan Lajka, Stepan Svacina, Martin Matoulek

**Affiliations:** Centre for Tobacco-Dependent of the 3rd Medical Department - Department of Endocrinology and Metabolism, First Faculty of Medicine, Charles University in Prague and General University Hospital in Prague, Karlovo namesti 32, 121 08 Prague 2, Czech Republic; Institute of Hygiene and Epidemiology, First Faculty of Medicine, Charles University and General University Hospital in Prague, Studnickova 7, 128 00 Prague 2, Czech Republic; STEM/MARK, a.s., Chlumčanského 497/5, 180 00 Praha 8, Czech Republic; 3rd Medical Department – Department of Endocrinology and Metabolism, First Faculty of Medicine, Charles University in Prague and General University Hospital in Prague, U Nemocnice 1, 121 08 Praha 2, Czech Republic

**Keywords:** Smoking, Hypertension prevalence, Smoking cessation

## Abstract

**Background:**

Several hypotheses suggest a temporary increase in blood pressure following smoking cessation. This may be the result of endocrine changes (e.g. alteration in adrenocorticotropic hormone and cortisol levels in post-cessation period) and/or post-cessation weight gain. Our aim was to identify factors that may be associated with the diagnosis of hypertension after quitting smoking.

**Methods:**

In 2010, we conducted a cross-sectional survey in a sample of 2065 Czech adults, chosen by quota selection and representative according to age, gender, education, region of residence and the size of settlement, aged 18 to 94 years. We examined the association between age, gender, body mass index, smoking status, and education with the hypertension diagnosis in their personal history. Data were compiled and weighed by age categories. Statistical significance was measured by Pearson Chi-square test at the level of significance 95 %.

**Results:**

Diagnosis of hypertension was reported in 461 (22 %) subjects, with no difference by gender. Based on univariate analysis, former smokers were more likely than non-smokers to be diagnosed for hypertension (OR 1.450 (1.110-1.900), *p* = 0.006). However, after adjusting for body mass index and age, the occurrence of hypertension diagnosis did not differ among non-smokers, smokers and former smokers (OR 0.760 for smokers, *p* = 0.082 and OR 1.020 for former smokers, *p* = 0.915).

**Conclusion:**

We did not find any differences in hypertension diagnosis prevalence according to smoking status.

## Background

Cardiovascular diseases (CVD) are the most common cause of death in the Czech Republic [1]. Smoking and hypertension are the most important preventable cardiovascular (CV) risk factors. Cigarette smoking leads to an immediate increase in blood pressure (BP), heart rate and myocardial contractility. These changes are largely due to the potent sympathomimetic effects of nicotine [2]. Smoking is known to alter hormone levels in both sexes [3, 4]. An increase in adrenocorticotropic hormone (ACTH), cortisol, aldosterone, angiotensin converting enzyme and catecholamine levels have been described [5]. Smoking also alters vasomotor functions due to impaired protease-activated receptor type 1 [6], increases arterial stiffness [7] and carotid artery wall thickness [8].

Some epidemiological studies have reported lower BP in smokers compared to nonsmokers [9]. In contrast, other studies have described higher levels of BP with 24-h ambulatory blood pressure monitoring (ABPM) in smokers, compared to non-smokers. Although, BP levels measured in the doctors‘office are almost the same [10–12].

There is conflicting evidence about the prevalence of hypertension among former smokers. Some studies reported an increase in blood pressure following smoking cessation [13–17], and others reporting no increase [18, 19] or even early reduction in blood pressure on cessation [20–22]. Various hypotheses including weight gain following smoking cessation and increased stress as a consequence of smoking cessation are provided as reasons for the observed increase in blood pressure after smoking cessation.

Our aim was to confirm or refute the hypothesis, that smoking cessation is associated with higher prevalence of hypertension diagnosis in personal history after adjusting for body mass index and age.

## Methods

In 2010, we conducted a cross-sectional survey in a sample of 2065 Czech adults aged 18 to 94 years. Data were collected as a part of a larger study titled "Live Healthy" aimed to examine the impact of dietary and exercise habits on overweight, obesity and related quality of life. Respondents were selected from a database of insured persons in the General Healthcare Insurance Company and were contacted via regular mail. The sample was collected by quota selection and is representative according to age, gender, education, region of residence and the size of settlement. Anyone 18 years or older who consented to participate in the project was eligible to participate. Data were collected in a 30–40 min, 68-item self-administered paper-pencil questionnaire handled by a trained interviewer. The response rate was not recorded. A copy of the questionnaire is available from the authors upon request. All the procedures were done in accordance with the Declaration of Helsinki (2000) of the World Medical Association.

### Study variables

We collected sociodemographic data including; age, gender, highest level of education achieved (basic = primary school or vocational education = apprenticeship certificate, without A level), vocational education with A level (=apprenticeship certificate + A level), A level versus university education), region of residence, the size of settlement, as well as a self-reported diagnosis of hypertension (i.e. diagnosed by physician according to ICD-10 [23]) and self-reported smoking status. Participants were divided into 3 groups according to smoking status: smokers (smoking currently at least 1 cigarette per day at the time of the survey), former smokers (used to smoke but had not smoked during the last month) and non-smokers (not smoking currently and had not smoked 100 or more cigarettes during their lifetime). Height was measured by sartorial tape (measuring tape), shoes off, after/prior to interview. Weight was measured on calibrated scale, light clothes, shoes off, prior/after to interview. For further analyses, subjects were divided into 4 categories according to age: 18–29, 30–44, 45–59 and 60+ and into 4 categories according to BMI (body mass index, kg/m^−2^): underweight (<18.500), normal weight (18.500–24.999), overweight (25.000–29.999) and obese (30.000 and more), which were merged into 2 categories (underweight + normal weight) when using logical regression (overweight + obese).

### Statistical analyses

Descriptive statistics were computed for the entire sample and then for males and females, separately, as well as for non-smoker, smokers and former smokers, separately. Data were compiled and weighed by age categories. Statistical significance was measured by Pearson Chi-square test at the level of significance 95 % (*p* = 0.050). For further statistical analysis univariate logistic regression was used to assess the risk of hypertension associated with smoking status, gender, age category, BMI category and education. Multivariable logistic regression considers theses variables simultaneously to assess the effect of smoking status on hypertension. Two models were used: model with adjustment all mentioned variables and model with adjustment only for BMI category. Software IBM SPSS statistics 21 was used for analysis. For all analysis *p* = 0.050 was considered statistically significant.

## Results

The questionnaire was completed by 2065 individuals, 49 % male. The mean age was 46.600 ± 17.700 (range 18–94). The mean age for males was 45.400 ± 17.300 years (range, 18–88 years) and 47.800 ± 18.000 years for females (range 18–94 years). In the sample, 461 (22 %) subjects reported the diagnosis of hypertension. The prevalence of the diagnosis of hypertension did not differ by gender (*p* = 0.341).

The majority of the sample, 1155 (56 %) were non-smokers, 562 (27 %) current smokers, and 348 (17 %) former smokers. The prevalence of smoking was higher among males than females (*p* < 0.001), see Table 1 for a summary of sociodemographic characteristics of the sample.

**Table 1:** Selected sociodemographic characteristics by gender and smoking status

Characteristic		Total	Males	Females	Non-smokers	Smokers	Former smokers
		(mean age 46.600 yrs SD 17.700)	(mean age 45.400 yrs SD 17.300)	(mean age 47.800 yrs SD 18.000)			
Number of subjects		2065	1014	1051	1155	562	348
		N (%)	N (%)	N (%)	N (%)	N (%)	N (%)
Age, years	18–29 yrs.	430 (20.800)	226 (22.300)	204 (19.400)	263 (22.800)**	127 (22.600)	40 (11.500)
	30–44 yrs.	557 (27.000)	284 (28.000)	273 (26.000)	317 (27.500)	169 (30.100)	71 (20.400)**
	45–59 yrs.	546 (26.400)	268 (26.400)	278 (26.500)	251 (21.700)	178 (31.700)**	117 (33.600)**
	≥60 yrs.	532 (25.800)	236 (23.300)	296 (28.200)	324 (28.100)**	88 (15.500)	120 (34.500)**
Highest education achieved	Without A-level	1203 (58.300)	611 (60.200)	592 (56.300)	645 (55.900)	361 (64.200)**	197 (56.600)
	With A-level	652 (31.600)	293 (28.900)	359 (34.200)**	385 (33.300)**	156 (27.800)	110 (31.900)
	University	203 (9.800)	107 (10.600)	96 (9.100)	119 (10.300)	45 (8.000)	39 (11.200)
	N/A	7 (0.300)	3 (0.300)	4 (0.400)	6 (0.500)	0 (0.000)	1 (0.300)
BMI category (kg/m-2)	Underweight (<18.500)	40 (1.900)	7 (0.700)	33 (3.100)**	29 (2.500)**	8 (1.400)	3 (0.900)
	Normal weight (18.500–24.999)	886 (43.000)	385 (38.000)	501 (47.700)**	520 (45.100)**	258 (45.900)	108 (31.000)
	Overweight (25.000–29.999)	701 (33.900)	406 (40.000)**	295 (28.100)	378 (32.700)	183 (32.600)	140 (40.200)**
	Obese (≥30.000)	438 (21.200)	216 (21.300)	222 (21.100)	227 (19.700)	113 (20.100)	97 (27.900)**
Blood pressure	Yes	461 (22.300)	217 (21.400)	244 (23.200)	266 (23.000)	90 (16.000)**	105 (30.200)
	No	1604 (77.700)	797 (78.600)	807 (76.800)	888 (76.900)	474 (84.200)	242 (69.500)
Smoking status	Non-smoker	1155 (55.900)	475 (46.800)	680 (64.700)**	X	x	x
	Smoker	562 (27.200)	335 (33.200)**	227 (21.500)	X	x	x
	Former smoker	348 (16.900)	203 (20.000)**	145 (13.800)	X	x	x

Statistical significance was measured by Pearson Chi-square test at the level of significance 95 % (*p* = 0.050) **significantly higher

Smokers were found to have lower levels of education than non-smokers (*p* = 0.024). We did not find significant differences in university graduation level among smokers and non-smokers. Non-smokers weighed less compared to former smokers and smokers (*p* < 0.001). 237 (68.1 %) former smokers were either overweight or obese compared to 296 (52.7 %) smokers and 605 (52.5 %) non-smokers. Former smokers tended to be older than non-smokers and smokers (*p* < 0.001) (Table 1).

Based on univariate analysis (Table 2), the prevalence of the diagnosis of hypertension was significantly associated with smoking status. Former smokers were less likely than non-smokers to be diagnosed by hypertension (OR 1.450 (95 % CI:1.110-1.900), *p* = 0.006). However, in the multivariate analysis after adjusting for gender, age, body mass index and education, the prevalence of hypertension diagnosis did not differ among non-smokers, smokers and former smokers (OR 0.760 for smokers, *p* = 0.082 and OR 1.020 for former smokers, *p* = 0.915).

**Table 2:** Odds ratio for hypertension among subgroups by smoking status, gender, age, body mass index and education

Variables	Univariate analyis		Multivariate analysis	
	(*N* = 2065, *N = 2057)		(*N* = 2057)	
	OR (95 % CI)^**^	*p* value^**^	OR (95 % CI)^**^	*p* value^**^
Smoking status				
Non-smoker	Reference	-	Reference	-
Smoker	0.620 (0.480–0.810)	<0.001	0.760 (0.560–1.040)	0.082
Former smoker	1.452 (1.110–1.900)	<0.006	1.020 (0.740–1.390)	0.915
Gender				
Male	Reference	-	Reference	
Female	1.110 (0.900–1.370)	0.322	1.040 (0.810–1.340)	0.757
Age category				
18–29 yrs.	Reference	-	Reference	-
30–44 yrs.	1.470 (0.790–2.730)	0.222	1.120 (0.600–2.090)	0.728
45–59 yrs.	8.810 (5.170–15.030)	<0.001	5.770 (3.340–9.960)	<0.001
60 and more yrs.	27.490 (16.250–46.520)	<0.001	17.400 (10.160–29.790)	<0.001
BMI category				
Normal weight + underweight	Reference	-	Reference	-
Overweight + Obese	5.350 (4.130–6.950)	<0.001	3.550 (2.660–4.730)	<0.001
Education*				
Basic school + vocational education (=missing A level)	Reference	-	Reference	-
A-level	0.680 (0.540–0.860)	<0.001	0.900 (0.680–1.180)	0.430
University	0.730 (0.500–1.060)	0.094	0.870 (0.560–1.340)	0.518

^**^OR - odds ratio reports measure of relative risk of hypertension in 95 % confidence interval. The *p*-value of Waldo test higher than 0.050 means that the cathegory does not belong to the model. Odds ratio higher than 1 means that the probability of hypertension increases in this cathegory

The diagnosis of hypertension was age-related. Using the category 18–29 years as a reference, it was significantly more often among those 45–59 years of age (OR 8.810 [95 % CI: 5.170-15.030], *p* < 0.001) and 60 + (OR 27.490 [95 % CI: 16.250-46.520], *p* < 0.001) in univariate analysis. This was also confirmed by multivariate analysis: for the age category 45–59 years with OR 5.770 (95 % CI: 3.340-9.960), *p* < 0.001 and age 60 + with OR 17.400 (95 % CI: 10.160-29.700, *p* < 0.001).

The diagnosis of hypertension was associated with BMI (Table 2) being present in 45 % of obese patients (197/438), in 26 % of overweight patients (184/701) and in 9 % of normal weight patients (79/886). Only 2 % of underweight patients had hypertension (1/40). Obese and overweight patients showed significantly higher prevalence of the diagnosis of hypertension (OR 5350 [95 % CI: 4.130-6.950, *p* < 0.001]), both for univariate and for multivariate analysis (Table 2).

Based on univariate analysis, the prevalence of the diagnosis of hypertension was significantly higher among groups with basic education + vocational education (=missing A-level), used as reference, and group with A-level (OR 0.680 [95 % CI: 0.540-0.860], *p* < 0.001). After adjusting for above mentioned parameters, no difference persisted (OR 0.900 [95 % CI: 0.680-1.180], *p* = 0.430). No difference was found among reference group and university education group (OR 0.730 (*p* = 0.094) for univariate and OR 0.870 (*p* = 0.518) for multivariate analysis (Table 2).

## Discussion

We found no difference in the prevalence of the diagnosis of hypertension according to smoking status in the Czech Republic (non-smokers, smokers and former smokers) after adjusting for body mass index and age, although univariate analysis found former smokers less likely to be hypertensive compared to non-smokers. Possible explanation of the univariate analysis result is weight-correlated presence of hypertension - former smokers weighed more compared to non-smokers. There was no difference in the prevalence of the diagnosis of hypertension among smokers after adjusting for age and body mass index.

Our findings agree with those of Halimi *et al.*, Onat *et al.* and Jazon *et al.* [15–17], which also found a difference in the prevalence of hypertension according to smoking status which is connected to BMI. Adjusting for age and BMI is important, because age is involved in the pathogenesis of hypertension [24] and the majority of former smokers gain weight after smoking cessation [25]. Post-cessation weight gain is multifactorial. Probably one of the most important causes is nicotine itself, because it increases the basal metabolic rate by up to 10 % via sympathetic stimulated thermogenesis and oxidation of fatty acids [26–28]. Additionally, Mineur et al. found nicotine mediated stimulation of proopiomelanocortin system (POMC) resulting in decrease of appetite in smokers [29].

In contrast, Lee et al. reported higher levels of both systolic and diastolic BP among those who had quit smoking for ≥ 1 year in a 4-year prospective study [22]. The authors monitored 8,170 male steel workers who were examined in 1994 and re-examined in 1998 [22]. Higher BP values in former smokers were similar to weight gainers as well as weight losers and maintainers. All data were adjusted for baseline BMI, age, alcohol consumption (grams per week), cigarette smoking (pack-years), exercise (times per week), family history of hypertension, systolic BP or diastolic BP (baseline for the dependent variable), as well as changes in BMI and alcohol consumption during the follow-up period. Stratified analyses based on weight changes during 4 years were included. One possible explanation is the ACTH increase during smoking abstinence [30]. Smoking damages the vessel wall, possibly increasing the synthesis of prostacyclin and enhances the interaction between platelets and vessel wall [31]. These changes lead to decreased aorta elasticity [32]. Increased arterial stiffness can persist up to 10 years after smoking cessation [33], and may possibly increase the prevalence of hypertension among former smokers.

We found that those suffering from hypertension tend to be older, as well as overweight or obese. A higher prevalence of hypertension in older adults is generally known, as well as the higher prevalence of hypertension among those being overweight or obese. It has been described, that overweight/obese patients suffer more often by chronic medical disorders including arterial hypertension [34].

In our sample, more smokers were male gender and had lower education level. This finding reflects current knowledge and present situation in the Czech Republic, with higher prevalence among those with lower socioeconomic status and among male gender [35].

Our data showed that former smokers were older compared to non-smokers and smokers. Older age among former smokers was noted also by other authors [36] and may be explained by decreasing smoking prevalence with rising age as a consequence of health problems and/or due to concerns about health. Age can also be perceived as a cumulative measure with a greater probability of older individuals compared to younger ones being former smokers. Another explanation is survival bias due to greater survival of former smokers when compared to individuals who continue to smoke [36].

We also found that non-smokers weighed less compared to former and current smokers. Higher BMI among former smokers compared to smokers and non-smokers corresponds with results of large population studies [33, 37] and is caused by post-cessation weight gain. In addition, large population studies also described lower BMI of smokers compared to non-smokers [18, 37–41]. As mentioned above, nicotine acts as anorectic by increasing the basal metabolism and decreasing appetite.

The limitation of the study is its cross sectional design. This type of study allows to estimate the percentage of sick persons as well as persons with risk factor in the population, but there is no possibility to determine whether exposure preceeded the disease or vice versa. Furthemore, cross-sectional design has the impact on the outcomes of the study due to lack of associations. Finally, it is important to acknowledge that some confounding factors may limit the study results. One can argue, that most differences between the groups of former and current smokers could be explained by the different age distribution, as former smokers are older in average [36]. Also, obese people with a diagnosis of hypertension may be more likely to quit, based upon the doctor’s recommendation. Moreover, smokers could be less likely to be diagnosed with hypertension, as they usually don’t visit their doctor or participate in routine blood pressure measurements as often as non-smokers, but such data for the Czech population are not available.

Another limitation was the self-report of diagnosis of hypertension, especially if we consider that not all patients suffering from hypertension are diagnosed, properly treated or reported the diagnosis. In the representative sample of Czech population, approximately 40 % of adult population aged 25–64 years suffered from hypertension, while almost 30 % of patients with hypertension did not know about this diagnosis [42]. Additionally, high proportion of patients knowing about the diagnosis of hypertension, are not treated adequately [42]. Finally, smoking status was self-reported as well, thus some smokers may pretend to be former smokers or non-smokers and vice versa.

In this cross-sectional survey, the response rate was not recorded. The sample was selected by a quota choice and is representative in the proportional representation of participants according to age, gender, education, region of residence and the size of settlement. It can be stated, that the sample representativeness is of a limited value. But, when compared to the Czech adult population, our sample seems to be highly representative.

Additionally, it is important to acknowledge, that these data reflect the situation in the Czech Republic and may not be applicable to other countries.

As the post-cessation weight gain may increase the risk of developing hypertension, the practical implication of this study is to prevent weight gain and proceed with BP measurements more frequently during and after smoking cessation.

## Conclusion

In conclusion, no difference in the prevalence of the diagnosis of hypertension according to smoking status after adjusting for age and body mass index was found in our work.

## Abbreviations

CVD: Cardiovascular disease
CV: Cardiovascular
BP: Blood pressure
ACTH: Adrenocorticotropic hormone
ABPM: Ambulatory blood pressure monitoring
BMI: Body mass index
POMC: Proopiomelanocortin system
CPD: Cigarettes per day
